# P-1163. The Efficacy and Safety of Tosufloxacin in the Treatment of Bacterial Infections in Children: Systematic Review

**DOI:** 10.1093/ofid/ofae631.1349

**Published:** 2025-01-29

**Authors:** Suyun Yong, Mengjie Yang, Mi Zhou

**Affiliations:** Department of Pharmacy, Shaanxi Provincial People's Hospital, XI'AN, Shaanxi, China (People's Republic); Children's Hospital of Soochow University, Suzhou, Jiangsu, China; Children's Hospital of Soochow University, Suzhou, Jiangsu, China

## Abstract

**Background:**

In recent years, the use of antibiotics in pediatric patients has garnered significant attention due to the rise in drug-resistant bacteria. Tosufloxacin(TFLX) was used in pediatric pneumonia, otitis media, cholera, and anthrax. However, the limited use of TFLX in children is attributed to its adverse effects on bones and muscles. This article retrospectively analyzed the effectiveness and safety of TFLX in treating bacterial infections in children.

**Figure:**
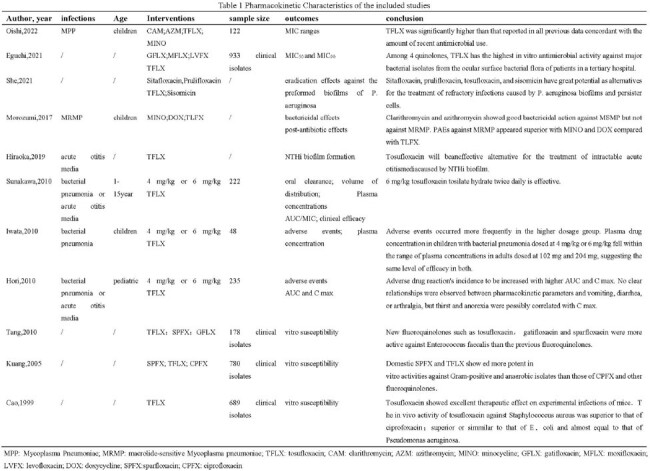

**Methods:**

A comprehensive search was conducted on PubMed, Web of Science, and Embase, up to March 2024. Data regarding the pharmacokinetics, efficacy, and safety of TFLX use in children were included.

**Figure:**
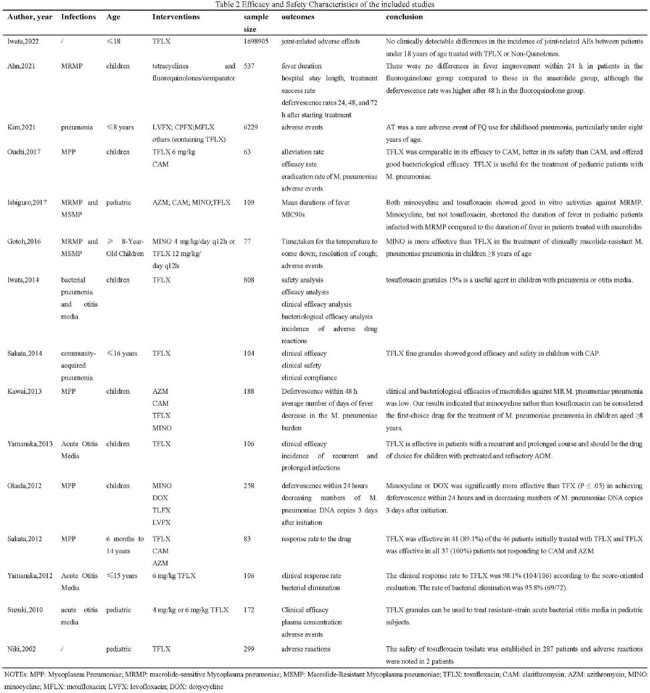

**Results:**

A total of 26 articles were involved, indicating that the pharmacokinetic outcomes of 222 children with infectious diseases were significantly correlated with their body weight following administration of 4 or 6mg/kg of TFLX. The incidence of adverse reactions was associated with AUC and C_max_ levels, where higher values of these parameters corresponded to increased adverse reactions. Notably, TFLX exhibited promising efficacy against MRMP, potentially surpassing macrolides, with minocycline showing superior efficacy compared to TFLX. Real-world data revealed a 100% clinical efficacy of TFLX in treating bacterial pneumonia in children. Moreover, a study involving 172 children with otitis media reported 97.7% and 94.1% clinical effectiveness rates for 4mg/kg and 6mg/kg doses of TFLX, respectively. Similar conclusions were drawn across various studies. Children under 2 years old, due to their functional and immune immaturity, benefitted from TFLX treatment for otitis media. The drug also demonstrated efficacy as a secondary option for children with refractory otitis media. Safety assessments highlighted diarrhea as the primary adverse reaction associated with TFLX, with minimal joint-related adverse effects. Some studies indicated a higher incidence of adverse reactions with 6mg/kg compared to 4mg/kg doses, suggesting a dose-dependent relationship. Overall, TFLX exhibited a good safety profile in pediatric infection management.

**Conclusion:**

The application of TFLX in children with pneumonia and otitis media may be safe and effective, but its efficacy in MRMP is weaker than that of minocycline.

**Disclosures:**

**All Authors**: No reported disclosures

